# Prediction of recurrence of early gastric carcinoma after endoscopic submucosal dissection

**DOI:** 10.4314/ahs.v23i2.32

**Published:** 2023-06

**Authors:** Hongjing Xu, Xiaojun Yang, Ying Lu, Shiming Wang

**Affiliations:** Department of Gastroenterology, Wuxi Xishan People's Hospital, Wuxi 214000, Jiangsu Province, China

**Keywords:** Endoscopy, gastric carcinoma, nomogram model, recurrence, submucosal dissection

## Abstract

**Background:**

The aim of this retrospective study was to predict the post-endoscopic submucosal dissection (ESD) recurrence risk of early gastric carcinoma (EGC) using a nomogram model, and to provide valuable evidence for preventing the recurrence.

**Methods:**

The patients with EGC receiving ESD between March 2010 and February 2018 were enrolled. They were followed up once every three months after ESD. At the end of follow-up, they were assigned into recurrence and non-recurrence groups. The independent risk factors for post-ESD recurrence were identified using Cox regression analysis. A nomogram prediction model was established, and its predictive efficiency was assessed by plotting receiver operating characteristic (ROC) curve.

**Results:**

*Helicobacter pylori* (HP) infection, number of positive lymph nodes >3 and a large amount of intraoperative hemorrhage were risk factors for post-ESD recurrence. According to multivariate Cox regression analysis, HP infection and number of positive lymph nodes >3 were independent predictors. The nomogram model had a good fitting effect, and the area under ROC curve was 0.933 (95% confidence interval: 0.919-0.947), suggesting a high predictive efficiency.

**Conclusion:**

Positive lymph nodes and HP infection are closely correlated with the recurrence risk after ESD in EGC patients. The established model is a quantitative tool for predicting recurrence to improve the prognosis.

## Introduction

As one of the clinically common digestive tract malignant tumors, gastric cancer ranks 2^nd^ among all tumors in terms of mortality rate, seriously affecting the life health and quality of life of patients^1^. Gastric cancer is characterized by insidious onset and rapid progression, and patients often have already been at middle and advanced stages when diagnosed, so early detection, diagnosis and treatment are crucial for improving the treatment outcomes and survival quality^2^. Early gastric carcinoma (EGC), which refers to a malignancy in the cancerous gastric mucosa or submucosa cells, is classified regardless of tumor size or lymph node metastasis^3^. EGC has been detected and resected more easily with the development of upper endoscopy recently, thus elevating the 5-year survival rate of over 90% cases with a minimal risk of lymph node metastasis^4^.

EGC patients are commonly treated through radical resection^5^, but this method leads to large surgical trauma, considerable intraoperative hemorrhage, obvious complications and digestive function impairment, thus severely affecting the postoperative recovery^6^. Besides, the cancer cells in EGC patients only invade the gastric mucosa and submucosa, so only cancerous gastric mucosal lesions need to be dissected for treatment. Endoscopic submucosal dissection (ESD) is a minimally invasive technique by which the cancerous gastric mucosa can be completely resected endoscopically^7^. At present, it is one of the major treatment methods for EGC due to the advantages of small trauma, mild stress response, few complications, rapid postoperative recovery, low recurrence rate and good prognosis^8^. ESD complete resection exerts an obviously better prognostic effect than ordinary ESD, but it is still difficult to avoid postoperative recurrence^9^.

Therefore, the risk factors for recurrence after ESD complete resection were herein screened for EGC patients, and a nomogram model that predicted recurrence was established to explore the possible causes of recurrence.

The objective of this study was to help formulate reasonable individualized diagnosis and treatment regimens, and to provide reliable data for the prevention and treatment of postoperative recurrence.

## Materials and Methods

### Subjects

In this retrospective study, the patients with EGC who received ESD in Wuxi Xishan People's Hospital between March 2010 and February 2018 were enrolled as the subjects. Inclusion criteria: The patients aged over 18 years old, undergoing ESD for the first time, diagnosed as EGC according to postoperative pathological examination, with indications of ESD and complete clinical data, and whose lesions were completely resected by surgery were included. Exclusion criteria: The patients undergoing phase 2 surgical treatment after ESD, or with concurrent malignant tumors, surgical contraindications, coagulation disorders, positive margin or base as shown in pathological examination, or incomplete data were excluded. Finally, a total of 408 subjects were enrolled. This study was reviewed and approved by the ethics committee of Wuxi Xishan People's Hospital, and all the subjects provided their informed consent and signed.

### Treatment methods

The patients were deprived of food and water 8 h before operation, subjected to general anesthesia by endotracheal intubation, and then placed in the left lateral position. The gastric lesion range and depth were confirmed by methylene blue staining combined with magnifying endoscopy. Subsequently, a dotted electrocoagulation marker was made using Dual knife electrosurgical knife (KD-650L) at 5 mm from the outer margin of the lesion, at which the mixture of glycerol, indigo carmine, adrenaline, fructose and NaCl was injected submucosally to achieve lesion bulge. Then the mucosa was cut open by IT knife (KD-611L) along the outer margin of the marker point, and the lesion was completely dissected along the submucosa, during which the above mixture was injected to facilitate dissection. Bleeding was stopped by nitrogen ion curing, electrocoagulation knife or thermal biopsy forceps (FD-410LR). In severe cases, the blood vessels were clamped using a titanium clip (Hx-610.135) and fixed by formaldehyde at the end of dissection.

### Collection of clinical data

The pre-ESD general data [age, sex, body mass index (BMI), history of smoking, and history of chronic diseases] and clinical data (diameter of tumors, degree of differentiation, depth of invasion, number of positive lymph nodes, position of tumors, surgical duration, amount of intraoperative hemorrhage, and resected area of lesions) were collected.

### Follow-up

The postoperative follow-up was conducted by endoscopy in the hospital once every three months from the initiation of ESD. If suspected lesions were endoscopically found, pathological examination was performed to observe whether there was recurrence. All subjects were followed up until recurrence or for 48 months in total. At the end of follow-up, the patients were assigned into recurrence group and non-recurrence group.

### Statistical analysis

SPSS 20.0 software and R 4.0.2 software were used for statistical analysis. The count data were expressed as [n (%)], and intergroup comparison was conducted the χ^2^ test. The normally distributed measurement data were represented as (-x ± s) and compared by the student's t test. Dichotomous Cox regression analysis was used for screening the predictors for death, and a nomogram prediction model was established using the package rms. Receiver operating characteristic (ROC) curves were plotted to analyse the ability of all indicators to predict cancer. The prediction model was validated by calibration curve and bias-corrected concordance index (C-index). P<0.05 suggested that a difference was statistically significant.

## Results

### General and clinical data of non-recurrence and recurrence groups

Of the enrolled 408 subjects, 362 (88.73%) were assigned into non-recurrence group and 46 (11.27%) were allocated into recurrence group. No statistically significant differences were found between the two groups regarding age, sex, history of smoking, concurrent hypertension, concurrent diabetes, gastric ulcer, tumor position, surgical duration, resected area of lesions, diameter of tumors, degree of tumor differentiation or depth of invasion, (P>0.05), but *Helicobacter pylori* (HP) infection, amount of intraoperative hemorrhage and number of positive lymph nodes were significantly different between the two groups (P<0.05) (Table 1).

**Table 1:** General and clinical data of non-recurrence and recurrence groups

Item	Non-recurrence group (n=362)	Recurrence group (n=46)	*t*/χ^2^	P
Age (n, %)			0.098	0.754
<65 years old	182(50.28)	22(47.83)		
≥65 years old	180(49.72)	24(52.17)		
Sex (n, %)			0.057	0.812
Male	190(52.49)	25(54.35)		
Female	172(47.5l)	21(45.65)		
BMI (n, %)			0.116	0.734
<24 kg/m^2^	195(53.87)	26(56.52)		
≥24 kg/m^2^	167(46.13)	20(43.48)		
History of smoking (n, %)			0.271	0.603
No	182(50.28)	25(54.35)		
Yes	180(49.72)	21(45.65)		
Concurrent hypertension (n, %)			0.372	0.542
No	80(22.10)	12(26.09)		
Yes	282(77.90)	34(73.91)		
Concurrent diabetes mellitus (n, %)			0.408	0.523
No	72(19.89)	11(43.91)		
Yes	290(80.11)	35(76.09)		
Gastric ulcer (n, %)			0.099	0.753
No	298(82.32)	37(80.43)		
Yes	64(17.68)	9(19.57)		
HP infection (n, %)			15.393	<0.001
Negative	290(80.11)	25(54.35)		
Positive	72(19.89)	21(45.65)		
Diameter of tumors (n, %)			0.098	0.754
≤2 cm	182(50.28)	22(47.83)		
>2 cm	180(49.72)	24(52.17)		
Degree of differentiation (n, %)			0.300	0.861
High differentiation	182(50.28)	22(47.83)		
Moderate differentiation	106(29.28)	13(28.26)		
Low differentiation	74(20.44)	11(23.91)		
Depth of invasion (n, %)			0.472	0.492
Intramucosal	254(70.17)	30(65.22)		
Submucosal	108(29.83)	16(34.78)		
Number of positive lymph nodes (n, %)			41.911	<0.001
<3	254(70.17)	10(21.74)		
≥3	108(29.83)	36(78.26)		
Position of tumors (n, %)			1.120	0.571
Gastric antrum	162(44.75)	22(47.83)		
Gastric angle	137(37.85)	14(30.43)		
Cardiac part and gastric fundus	63(17.40)	10(21.74)		
Surgical duration (min, χ̅ ± s)	58.32±8.12	60.46±11.28	1.603	0.110
Amount of intraoperative hemorrhage (mL, x̅ ± s)	65.12±5.43	69.89±6.24	5.515	<0.001
Resected area of lesions (mm^2^, χ̅ ± s)	35.33±7.12	34.92±7.23	0.367	0.714

BMI: body mass index, HP: *Helicobacter pylori*.

### Cox regression analysis results of post-ESD recurrence in EGC patients

The univariate Cox regression analysis results showed that HP infection, number of positive lymph nodes >3 and a large amount of intraoperative hemorrhage were the risk factors for post-ESD recurrence in EGC patients. According to multivariate Cox regression analysis results, HP infection and number of positive lymph nodes >3 were the independent predictors for post-ESD recurrence in EGC patients (Table 2).

**Table 2:** Univariate and multivariate Cox regression analysis results of post-ESD recurrence in EGC patients

Variable	Univariate analysis		Multivariate analysis	

	HR (95% CI)	P	HR (95% CI)	P
Age (year)				
<65	-	-		
≥ 65	1.211(0.908-1.543)	0.087		
Sex				
Male	-	-		
Female	0.912(0.824-1.156)	0.259		
BMI (kg/m^2^)				
<24	-	-		
≥24	0.823(0.654-1.178)	0.124		
History of smoking (n, %)				
No	-	-		
Yes	1.211(0.821-1.780)	0.332		
Concurrent hypertension				
No	-	-		
Yes	1.023(0.812-1.246)	0.122		
Concurrent diabetes mellitus				
No	-	-		
Yes	1.113(0.914-1.267)	0.079		
Gastric ulcer				
No	-	-		
Yes	1.257(0.923-1.782)	0.343		
HP infection				
Negative	-	-	-	-
Positive	2.56(2.123-3.432)	<0.001	2.113(1.324-2.768)	<0.001
Tumor diameter				
≤2 cm	-	-	-	-
>2 cm	1.121(0.911-1.345)	0.089		
Degree of differentiation				
High differentiation	-	-		
Moderate differentiation	1.023(0.843-1.346)	0.123		
Low differentiation	1.211(0.912-1.489)	0.078		
Depth of invasion (n, %)				
Intramucosal	-	-		
Submucosal	1.134(0.823-1.356)	0.189		
Number of positive lymph nodes				
<3	-	-	-	-
≥ 3	5.221(3.911-6.678)	<0.001	4.342(4.011-5.767)	<0.001
Position of tumor				
Gastric antrum	-	-		
Gastric angle	0.911(0.827-1.082)	0.123		
Cardiac part and gastric fundus	0.923(0.785-1.045)	0.228		
Surgical duration (min)	1.311(0.973-1.679)	0.097		
Amount of intraoperative hemorrhage (mL)	1.532(1.186-1.845)	0.022	1.011(0.812-1.328)	0.207
Resected area of lesions (mm^2^)	0.923(0.724-1.556)	0.256		

BMI: body mass index, HP: *Helicobacter pylori*.

### Establishment of nomogram model predicting post-ESD recurrence in EGC patients

**Figure 1:**
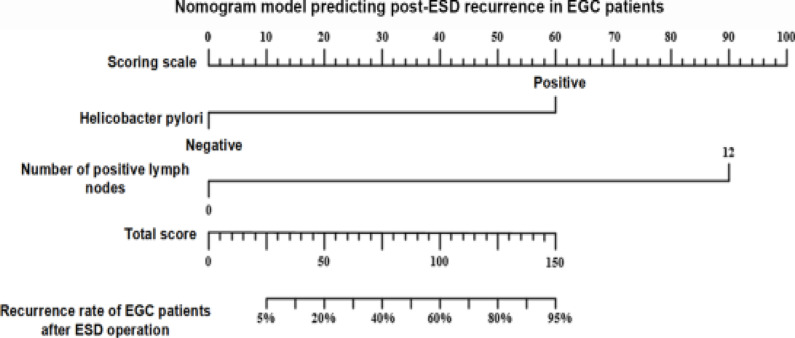
Nomogram model predicting post-ESD recurrence in EGC patients

A nomogram model was established using the independent predictive factors obtained from multivariate Cox regression analysis as the predictors. The length of line segments corresponding to the predictors in the nomogram represented the ability of the factors to predict post-ESD recurrence. The position of corresponding line segments of all the predictors was confirmed on the scoring scale according to the actual conditions of patients, and then each predictor was given a score. The scores of all the predictors were summed up, and the total score was positioned at the total score axis. The corresponding risk coefficient reflected the post-ESD recurrence risk. The number of positive lymph nodes was better than HP infection for predicting the post-ESD recurrence risk in EGC patients (Figure 1).

### Evaluation of accuracy and validity of nomogram model

**Figure 2:**
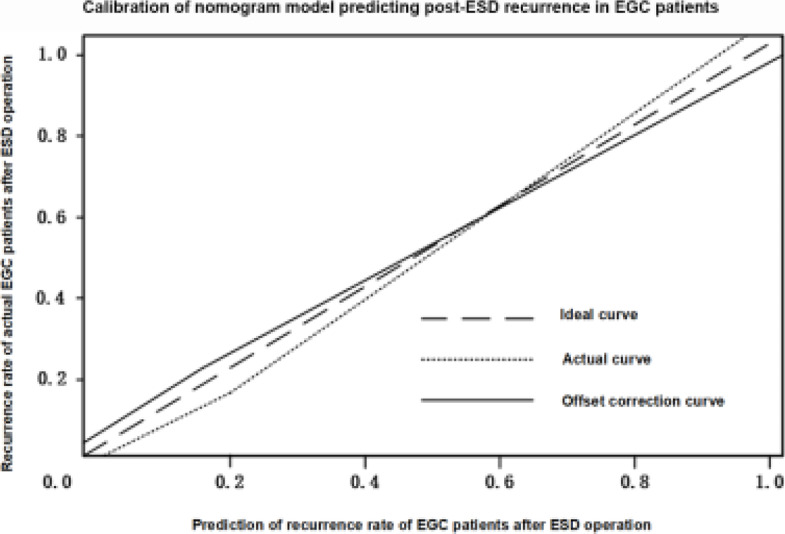
Calibration of nomogram model predicting post-ESD recurrence in EGC patients

The calibration and validity of the established nomogram model were assessed (Figure 2). The calibration index C-index was 0.916 (95% CI: 0.882-0.951), and the actual curve fitted well with the ideal one, suggesting that the predicted post-ESD recurrence using the model was consistent with the actual condition.

### ROC curve analysis results of nomogram model

**Figure 3:**
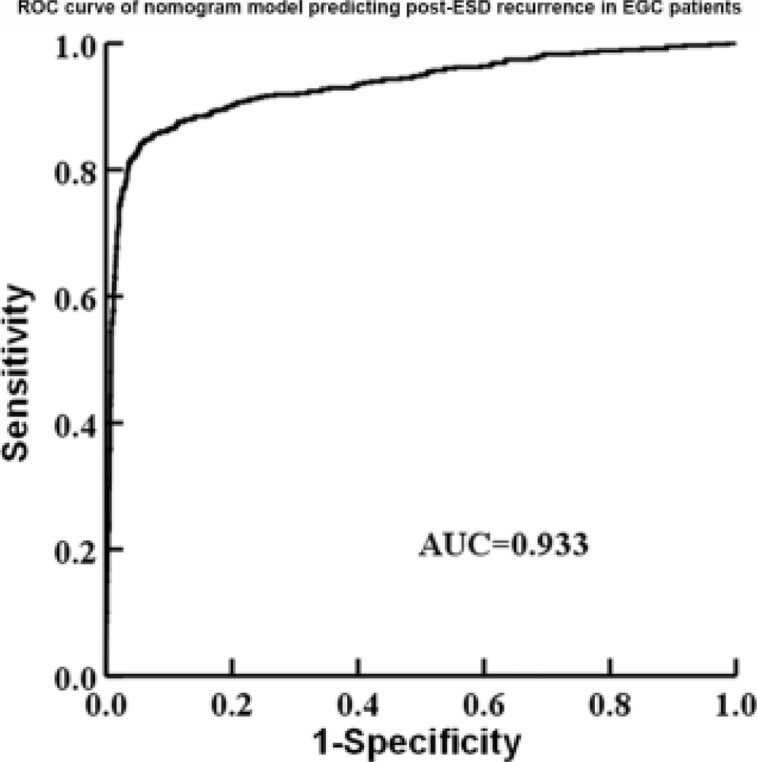
ROC curve of the nomogram model predicting post-ESD recurrence in EGC patients

The ROC curve was plotted to predict the post-ESD recurrence in EGC patients. The area under the ROC curve (AUC) was 0.933 (95% CI: 0.919-0.947), indicating that the model has a high predictive efficiency (Figure 3).

## Discussion

In recent years, the detection rate of EGC has been considerably raised with the rapid development of medical technologies, enhancement of public health awareness and popularization of physical examination^10^. Minimally invasive endoscopic surgeries, such as endoscopic mucosal resection (EMR)^11^ and ESD, have been gradually applied in the treatment of EGC. Although ESD is developed based on EMR, it allows en bloc resection of EGC tumors using high-frequency electric knife and auxiliary devices, also relieving patients' pain, promoting postoperative recovery, and further improving the quality of life and prognosis^12^.

In this study, the established nomogram model revealed that the number of positive lymph nodes had high predictive efficiency, followed by HP infection. The appearance of positive lymph nodes suggests the metastasis of gastric cancer, and the patients with more positive lymph nodes have severer conditions and higher risks of residual potential cancer cells in the lymphatic system and recurrence of gastric cancer. Additionally, the patients with positive lymph nodes also need lymph node dissection, and the dissection range is positively correlated with the number of positive lymph nodes, thus affecting the postoperative recovery and raising the recurrence risk of gastric cancer^13^. HP infection itself is an independent risk factor for gastric cancer^14^. In this study, HP infection was also a predictor for the recurrence in EGC patients, probably due to the following reasons. Firstly, it damages DNA in gastric mucosal cells to induce mutation, thereby generating cancer cells^15^. Secondly, long-standing inflammatory irritation leads to abnormal protein expression in gastric epithelial cells, thereby disturbing the proliferation-apoptosis balance and ultimately causing cancer^16^. Thirdly, nitrate is catalytically reduced to carcinogenic nitrite^17^. Fourthly, HP infection causes immune dysfunction by miRNAs, thereby inducing the recurrence of gastric cancer^18^. Fifthly, HP infection promotes the apoptosis of tumor necrosis factor-related apoptosis-inducing ligand, thus augmenting the recurrence risk of gastric cancer^19^. HP has long been confirmed to be a predictor for the postoperative recurrence in patients with gastric cancer^20^, but the underlying mechanism remains largely unknown.

In conclusion, positive lymph nodes and HP infection are the independent risk factors for post-ESD recurrence in EGC patients. The nomogram model based on these two predictors had high predictive efficiency, and AUC was 0.933 (95% CI: 0.919-0.947), so it can help develop individualized treatment regimens for EGC patients, thereby improving the prognosis. Regardless, this study has limitations. The sample size was small, and this was a single-center study. Besides, long-term follow-up was not conducted to observe the prognosis of patients due to limited research time. Therefore, multicenter studies with larger sample sizes and extended follow-up time are in need to validate the findings.
